# Neural alpha oscillations and auditory steady-state responses during adaptation to a cochlear implant

**DOI:** 10.1093/cercor/bhaf244

**Published:** 2025-09-10

**Authors:** Malte Wöstmann, Hannah Marie Meineke, Rainer Schönweiler, Daniela Hollfelder, Karl-Ludwig Bruchhage, Anke Leichtle, Jonas Obleser

**Affiliations:** Department of Psychology, University of Lübeck, Ratzeburger Allee 160, Lübeck 23562, Germany; Center of Brain, Behavior, and Metabolism, University of Lübeck, Ratzeburger Allee 160, Lübeck 23562, Germany; Department of Psychology, University of Lübeck, Ratzeburger Allee 160, Lübeck 23562, Germany; Center of Brain, Behavior, and Metabolism, University of Lübeck, Ratzeburger Allee 160, Lübeck 23562, Germany; Department of Clinical Research, University of Southern Denmark, Campusvej 55, Odense 5230, Denmark; Department of Otorhinolaryngology, Phoniatrics and Paediatric Audiology, University Hospital of Schleswig-Holstein, Campus Lübeck, Ratzeburger Allee 160, Lübeck 23562, Germany; Department of Otorhinolaryngology, Head and Neck Surgery, University Hospital of Schleswig-Holstein, Campus Lübeck, Ratzeburger Allee 160, Lübeck 23562, Germany; Department of Otorhinolaryngology, Head and Neck Surgery, University Hospital of Schleswig-Holstein, Campus Lübeck, Ratzeburger Allee 160, Lübeck 23562, Germany; Department of Otorhinolaryngology, Head and Neck Surgery, University Hospital of Schleswig-Holstein, Campus Lübeck, Ratzeburger Allee 160, Lübeck 23562, Germany; Department of Psychology, University of Lübeck, Ratzeburger Allee 160, Lübeck 23562, Germany; Center of Brain, Behavior, and Metabolism, University of Lübeck, Ratzeburger Allee 160, Lübeck 23562, Germany

**Keywords:** attention, auditory, cochlear implant, electroencephalography, temporal coding

## Abstract

The human auditory system must distinguish relevant sounds from noise. Severe hearing loss can be treated with cochlear implants (CIs), but how the brain adapts to electrical hearing remains unclear. This study examined adaptation to unilateral CI use in the first and seventh months after CI activation using speech comprehension measures and electroencephalography recordings, both during passive listening and an active spatial listening task. Neural phase-locking to amplitude-modulated sounds interacted with time, such that phase-locking longitudinally increased stronger for 40 Hz compared with 4 Hz. In the spatial listening task, the benefit of performing the task with the CI on vs. off was most pronounced when the CI ear was primarily exposed to target speech. Lateralized alpha oscillations (~10 Hz) reliably marked CI users’ focus of spatial attention. Stronger alpha modulation in the hemisphere opposite to the nonimplanted ear indicates an attentional bias toward the acoustically hearing ear. Our findings suggest that adaptation to hearing with a CI is accomplished by dynamic changes in auditory phase locking and a bias in auditory spatial attention.

## Introduction

Hearing loss is a common health condition that affects a large group of individuals above the age of 60 years (>65% according to WHO 2021). In many cases, severe hearing loss can be treated with the implantation of a cochlear implant (CI), which transduces acoustic energy into electrical signals that stimulate the auditory nerve (Eshraghi et al. 2012). Unilateral CI users with combined electrical hearing on one ear through the CI and acoustic hearing on the contralateral ear pose a relevant test case for two reasons. First, there is a relatively large number of unilaterally implanted individuals, and we do at present not sufficiently well understand how the brain adapts to altered sensory perception following unilateral cochlear implantation. Second, models of auditory attention are based in large parts on findings in normal-hearing listeners with symmetrical hearing. Unilateral CI use offers a model for probing how a healthy, fully functioning neural system adapts to the unilateral restoration of degraded sensory input and its effects on auditory spatial attention allocation.

Successful hearing and communication rest on the interlocking of specific neural mechanisms. Accurate sound encoding and auditory perception aid auditory object formation, whereas the allocation of auditory attention to enhance neural representations of target sounds and to suppress noise aids auditory object selection (Shinn-Cunningham 2008). In CI users, listening performance on average increases during the first 6 months after cochlear implantation, followed by a plateau (eg Lenarz et al. 2012). With the present study, we aim to investigate the neural dynamics of auditory perception and spatial attention in unilateral CI users during the first half year following cochlear implant activation.

Sound processing through a CI primarily degrades spectral cues but leaves temporal envelope cues largely intact. Thus, CI users are thought to rely mainly on temporal-envelope information (Rosen 1992; Shannon et al. 1995; Heng et al. 2011; Dincer D’Alessandro et al. 2018). Here, we ask how adaptation to hearing with a CI is reflected in neural phase-locking to the sound envelope. In the human electroencephalogram (EEG), the auditory steady-state response (ASSR), also referred to as the envelope following response (EFR) in case of a periodic envelope, has been linked to auditory temporal processing acuity (Roß et al. 2002; Purcell et al. 2004). ASSRs evoked by 40 Hz modulation have a particularly high SNR (Galambos et al. 1981) and are generated in auditory cortical and subcortical regions (Farahani et al. 2017; Luke et al. 2017; David et al. 2023), whereas ASSRs evoked by slower frequencies (<20 Hz) originate primarily from the auditory cortex (Liégeois-Chauvel et al. 2004). The 40 Hz ASSR is altered in central brain disorders (eg Kwon et al. 1999; Wilson et al. 2007; Grent-‘t-Jong et al. 2023) and has been associated with cortical inhibition (eg Toso et al. 2024).

In CI users, 4 and 40 Hz ASSRs relate to modulation detection thresholds, indicating their feasibility to assess temporal coding in CI users (Luke et al. 2015). Somewhat unintuitively, larger cortical representations of slow amplitude modulations (~4 Hz) have been observed at older age (Presacco et al. 2016; Anderson et al. 2020) and in hearing loss (Fuglsang et al. 2020; Orf et al. 2025), which might suggest an imbalance of excitatory and inhibitory processing. Here, we test specifically how adaptation to hearing with a CI reflects in changes of the 4 versus 40 Hz ASSR.

A prominent neural signature of spatial attention in different sensory modalities is the hemispheric lateralization of ~10 Hz alpha oscillatory power (auditory: Ahveninen et al. 2013; somatosensory: Haegens et al. 2011; visual: Worden et al. 2000). In normal-hearing listeners, alpha power increases in the hemisphere ipsilateral to the focus of auditory attention and decreases in the contralateral hemisphere (eg Müller and Weisz 2012; Wöstmann et al. 2016; Deng et al. 2020). Relatively reduced versus enhanced alpha power is thought to reflect the attentional selection of targets and suppression of distraction, respectively (eg Strauß et al. 2014; Schneider et al. 2021; Bonnefond and Jensen 2024). Through experimental separation of lateral target versus distractor processing, we have recently shown that there are two lateralized alpha responses (Wöstmann et al. 2019), one related to the selection of targets and another to the suppression of distraction (for related findings in the visual modality, see Yang et al. 2024; Cruz et al. 2025).

Evidence for auditory attentional alpha power modulation in CI users is scarce, however. Alpha power modulation in CI users has been found to relate to the subjective experience of task difficulty, ie listening effort (Dimitrijevic et al. 2019). Furthermore, in bilateral CI users, Paul et al. (2020) found evidence for alpha lateralization during auditory spatial attention to speech. However, we do not at present understand how asymmetric hearing loss and adaptation to hearing with a unilateral CI affect large-scale neural network organization (see also Jiwani et al. 2021) and the allocation of auditory spatial attention.

Here, we present data from a longitudinal EEG study in unilateral CI users to probe neural signatures of auditory perceptual processing and spatial attention allocation in the first and seventh months following CI activation. We asked, first, whether the relative pattern of slow (4 Hz) versus fast (40 Hz) ASSR responses would reflect a change in the excitation–inhibition balance during adaptation to listening with a CI. Second, we tested whether unilateral CI users exhibit asymmetry in the neural allocation of spatial attention depending on the side of the CI.

## Methods

### Participants

A total of *n* = 20 native German speakers were recruited for this study. Two participants dropped out before finishing the first recording session. Three participants dropped out of the study after completing the first session. Part of the EEG data of one participant were not recorded due to technical issues. Thus, *n* = 18 participants with complete data of the first session and *n* = 14 complete datasets with two recording sessions were available for analysis. Of these *n* = 14 participants with complete datasets, all but one participant were right-handed according to the Edinburgh handedness questionnaire (Oldfield 1971).

All participants were unilaterally fitted with a CI at the Department of Otorhinolaryngology, Head and Neck Surgery, University Hospital of Schleswig-Holstein, Campus Lübeck, Germany, with a CI from the manufacturers Cochlear or MedEL. Their contralateral hearing loss varied from normal hearing to profoundly hard of hearing. Participants were financially compensated for their participation at an hourly rate of €10. All participants gave written informed consent to participate in the study. All procedures were approved by the ethics committee of the University of Lübeck, Lübeck, Germany (ID of approval: 19–127).

### Study timeline and setup

Participants completed the same test battery twice: once approximately in the first month after activation of the CI (session 1) and once approximately six months thereafter (session 2). Each session comprised two data recordings on two separate days. Time intervals provided below refer to the second data recording of each session, which included the main tasks reported here (ie passive and active listening tasks in the EEG). For the *n* = 14 complete datasets, the average time interval between CI activation and session 1 was 3.3 weeks (range: 1.3 to 7.3). The average interval between sessions 1 and 2 was 6.4 months (range: 5.5 to 7.8).

Data recording spanned the time window from September 2020 to May 2023. Sound stimuli were presented via loudspeakers (Genelec 8020D), placed at a distance of ~70 cm from the participant’s head. Depending on the task and experimental condition, loudspeakers were positioned on the left side (−90°), right side (+90°), or in the front (0°). Individual tests (amplitude modulation rate discrimination [AMRD], passive listening, spatial attention task) were implemented in Matlab (R2017b) and Psychtoolbox (Brainard 1997).

In addition to the individual tests of the test battery explained below, participants performed a continuous speech tracking task (adapted from Kraus et al. 2021), which was not analyzed for the purpose of the present study.

### Audiological tests

Pure tone audiograms (at frequencies 0.125, 0.25, 0.5, 1, 2, 3, 4, 6, and 8 kHz) and speech intelligibility scores in the “Freiburg Monosyllabic Speech Test” (FMST, 65 dB SPL, sound pressure level) were collected prior to CI implantation and at the regular clinical appointment of the participant. The audiological measurements were conducted for both ears separately, if possible. The pre-operative audiogram and FMST were measured unaided; the postoperative measurements were conducted aided with the CI. The contralateral ear measurements were conducted, aided by a hearing aid when applicable.

Temporal sensitivity was assessed with an adaptive AMRD test (for details, see Erb et al. 2019). In a Three-Alternative Forced Choice (3-AFC) adaptive staircase paradigm, three broadband noise sounds were presented in sequence on each trial. Two standard stimuli were amplitude-modulated (AM) at 4 Hz, and one deviant stimulus was AM at a frequency of 4 to 6 Hz (determined according to the adaptive staircase method). The task always started with a 6 Hz deviant. The position of the deviant varied randomly on a trial-by-trial basis. Participants had the task to report the position (1 to 3) of the deviant via button press. A 2-down, 1-up adaptive staircase procedure determined the AMRD threshold, converging on 70.7% correct responses (Levitt 1971). The initial step size was 0.5 Hz, which was reduced to the minimal step size of 0.25 Hz after four reversals. The test terminated after 10 reversals. The AMRD threshold corresponded to the average AM rate difference of the deviant vs. standard across the last six reversals. Participants completed the AMRD task twice; the minimum of the two resulting thresholds was used for further analyses.

Participants filled out the short form of the “Speech, Spatial and Quality of Hearing Scale” (SSQ, German short version; Kießling et al. 2011) at the beginning of each of the two sessions to assess their subjective hearing performance (Gatehouse and Noble 2004). As an outcome measure, we used the sum of scores across all items, with a higher score corresponding to better self-rated hearing.

### Threshold estimation procedure

As absolute hearing levels differ dramatically between CI users, we aimed to present sound stimuli at an overall intensity that was adapted to individual hearing thresholds. To this end, absolute hearing thresholds were assessed using the method of limits. We used the same spoken numbers (1 to 9, female voice) as for the spatial attention task. For each speaker location (front, left, right), spoken numbers were presented in random order, starting at an inaudible intensity and increasing in steps of 1 dB. Participants were instructed to press a button as soon as they could hear a number. This procedure was repeated three times. Afterward, three repetitions of the same procedure followed with the exception that presentation started far above threshold and decreased in steps of 1 dB. Participants were instructed to press a button as soon as they could no longer hear the numbers anymore. The threshold was determined by averaging across scores of the last two repetitions for increasing and the last two repetitions for decreasing sound intensity. Thresholds for the front speaker location were always measured first, followed by the non-CI side and CI side. Thresholds varied considerably across participants and conditions (range: 43.75 dB), but a repeated-measures analysis of variance (ANOVA) revealed no main or interaction effects of speaker location (front, left, right) or session (1 vs. 2) on thresholds (all *P* > 0.33).

Determined thresholds were considered to correspond to 0 dB sensation level (SL) and sound stimuli in the passive listening task and spatial attention task were presented at +40 dB SL at each individual loudspeaker position. Separate threshold measurements were conducted in session 1 and session 2. Alternatively, it would have been possible to use participants’ pure tone thresholds for individual stimulus adjustments, especially for the passive listening task with AM sounds. However, since thresholds estimated from our procedure were highly correlated with pure tone thresholds and since the assessed ASSR in the passive listening task did not significantly relate to pure tone thresholds (see Fig. S3), there is no indication of bias in our threshold estimation procedure.

### Passive listening to AM sounds

Participants listened passively to 2-s long 1000 Hz tones, with three different AM rates: 4, 20, and 40 Hz (100% modulation depth). Sounds were presented binaurally at +40 dB SL over the left and right loudspeakers. In total, 180 sounds were presented (60 per AM rate) in a pseudo-randomized order, making sure no more than three consecutive sounds would have the same AM rate. The interstimulus interval was randomly jittered in 100-ms steps between 500 and 1,500 ms.

Of note, the 20 Hz AM rate was not of primary interest in the analysis of the ASSR. Instead, this condition was used in the experiment to increase the ability to separate auditory neural components (which should exhibit stronger phase-locking to 4 & 40 Hz compared with 20 Hz) from components related to the processing in the cochlear implant (which should exhibit similar phase-locking across frequencies).

### Active listening in an auditory spatial attention task

The design of the auditory spatial attention task was adapted from Wöstmann et al. (2019). The overarching aim was to implement listening conditions that separate attentional selection from suppression of lateralized sounds on the CI versus non-CI side. To this end, two competing spoken numbers were presented from two loudspeaker positions. One loudspeaker was always positioned in the front (0°), while the other changed its position from left (−90°) to right (+90°) in a block-wise fashion. This way, the following four conditions were implemented: *select CI-side, select non-CI-side, suppress CI-side,* and *suppress non-CI-side*.

On each trial, participants were first presented for 500 ms with a spatial cue (∧, <, or >) to indicate the location of the upcoming target sound (ie front, left, or right). In the ensuing anticipation period, randomly jittered in duration between 1,000 and 1,800 ms in steps of 100 ms, a fixation cross was presented on the screen. Then, two different, randomly selected numbers were presented, each one over one of the two loudspeakers. The set of numbers (adapted from Obleser et al. 2012) comprised the numbers 1 to 9, which were spoken by a female voice and adjusted to a duration of 500 ms each. To enhance the perception of simultaneous onsets of the two numbers on each trial, we applied a perceptual onset matching (as described in Wöstmann et al. 2016). Participants used a numpad to indicate the target number at the end of each trial.

The task comprised six blocks. Loudspeakers were positioned in the front and on the left in half of the blocks and in the front and on the right in the other half. Each block contained 50 trials, 25 with an attention cue to the front and 25 with a cue to the side (left or right). The loudspeaker setup (front & left versus front & right) alternated from block to block, starting with the front & left setup for participants with the CI on the right side and vice versa for participants with the CI on the left side. In total, the task contained 300 trials with 75 trials per experimental condition. During each block, continuous broadband background noise (band pass–filtered between 0.1 and 8 kHz) was presented from an additional loudspeaker positioned behind the participant at a low intensity of +25 dB (SL; as determined for the front loudspeaker in the threshold estimation procedure).

The task was implemented as an adaptive 1-down, 1-up staircase procedure (Levitt 1971) targeting the 50% speech reception threshold (SRT_50_). For each of the four experimental conditions, the task started at an SNR of +10 dB (except for the first participant in the first session, for whom it started at 0 dB), meaning that the target intensity was 10 dB higher than the distractor intensity. Following a correct response in one condition, the SNR was lowered by 2 dB for the next trial in this condition. Following an incorrect response in one condition, the SNR was increased by 2 dB for the next trial in this condition. The threshold in each condition was calculated by averaging the SNR for all trials except for the first block in which this condition was implemented. In session 1, three participants had partially missing behavioral data (last two blocks in CI off recording missing for ID 4 & 5; last block in CI off recording missing for ID 7).

In each session (1 & 2), each participant performed the auditory spatial attention task twice, first with the CI switched on and then, after a short break, with the CI switched off.

### E‌EG preprocessing and CI artifact detection

The EEG was recorded at 64 active scalp electrodes (Ag/Ag-Cl; ActiChamp, Brain Products) at a sampling rate of 1000 Hz, with a DC–280 Hz bandwidth, against reference electrode Fz. All electrode impedances were kept below ~30 kΩ. Note that, depending on the placement of the cochlear implant (and the hearing aid on the contralateral side), some electrodes above the device could not be connected and were interpolated later (see below). To ensure equivalent placement of the EEG cap, the vertex electrode (Cz) was placed at 50% of the distance between inion and nasion and between left and right ear lobes. For EEG data analysis, we used the FieldTrip toolbox (Oostenveld et al. 2010) for MATLAB (R2013b/R2023b) and a custom script.

**Fig. 1:**
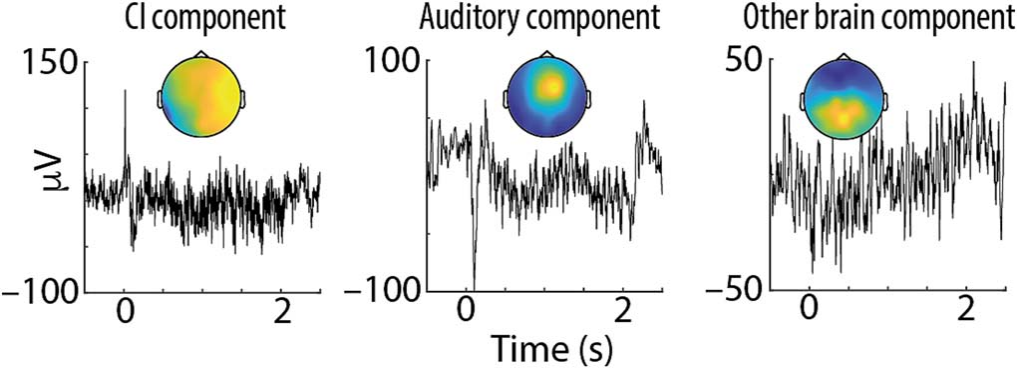
Time courses (averaged across all trials and AM rates in the passive listening task) and topographic maps of exemplary ICA components for one participant with the CI on the right side (ID 5, session 1). The CI artifact component shows a sharp, low-latency response to sound onset and largest weights on the CI side. The auditory component has the highest weights at central electrode sites and shows typical auditory onset and offset responses in the beginning (0 s) and end (2 s) of sound presentation, respectively. The “other brain component” depicted here has high weights at parietal electrodes, as it is typical for alpha (~10 Hz) oscillatory power.

We used the data from the passive listening task to identify independent components in the EEG data relating to artifacts, auditory activity, and other brain activity for further analyses. To this end, the continuous data were filtered (high pass: 0.1 Hz, low pass: 100 Hz) and split into epochs around sound onsets (−0.5 to 2.5 s). Next, trials for which any channel exceeded a range of 300 mV in the time interval 0 to 2 s were removed. An independent component analysis (ICA) was used to split the data into a number of independent components required to explain 90% variance. Component time courses, topographic maps, and frequency spectra were carefully inspected (see Fig. 1) to identify (i) components relating to the CI artifact (sharp, low-latency sound-evoked activity, largest at electrodes close to CI), (ii) components relating to auditory neural processing (sound-evoked pattern of P1-N1-P2 components, strongest at central electrodes), (iii) other brain components (components with typical characteristics of electrophysiological brain activity, such as 1/f spectra), and (iv) remaining artefactual components (relating, eg to eye-blinks or saccades, muscle activity, or line noise). Due to the characteristic differences of components related to CI processing versus auditory neural activity in topography and latency, CI-artifact removal from the EEG data was possible in the present dataset.

### Analysis of EEG data in the passive listening task

Continuous EEG data of the passive listening task were filtered (high pass: 0.1 Hz, low pass: 100 Hz) and split into epochs around sound onsets (−0.5 to 2.5 s). To focus on auditory activity, we projected the data through the previously identified auditory components from each participant (separately for data in sessions 1 and 2). Next, missing channels were interpolated (spline interpolation) and the data were re-referenced to the average of all channels. Epochs with the 10% largest ranges (in time interval 0 to 2 s; at any channel) were removed.

To quantify the magnitude of the neural onset response to amplitude modulated sound stimuli, we quantified global field power (GFP) separately for each session (1, 2), participant, and AM rate (4, 20, 40 Hz) by calculating the standard deviation of EEG activity across electrodes in the time interval spanning obligatory onset responses (P1, N1, & P2; 0 to 230 ms).

To analyze the ASSR, epochs were split according to the three AM rates (4, 20, 40 Hz) and the event-related potential (ERP) was calculated by averaging over trials in the time domain. To focus on activity unrelated to the sound onset-evoked response, we cut out ERPs in the time interval 0.5 to 2 s and then obtained spectral power using Fast Fourier Transform (FFT; rectangular window) for frequencies 1 to 60 Hz in steps of 1 Hz with 2 Hz spectral smoothing. Finally, spectral power was averaged over nine fronto-central electrodes (Fz, F1, F2, FCz, FC1, FC2, Cz, C1, C2).

### Analysis of EEG data in the spatial attention task

Continuous EEG data of the auditory spatial attention task were filtered (high pass: 1 Hz, low pass: 100 Hz) and split into epochs around visual cue onset (−1 to 4 s). We used the previously identified ICA components (see above), projected the data through these components, and rejected CI and other artifactual components (separately for data in sessions 1 and 2). Missing channels were interpolated (spline interpolation), and the data were re-referenced to the average of all channels. Epochs with the 10% largest ranges (in time interval 0 to 2.5 s; at any channel) were removed. Finally, epochs were split into experimental conditions. For three participants in session 1 (ID 4, 5, 16), one block of EEG data was missing and thus not included in the analysis.

Based on previous studies in healthy, normal hearing participants (eg Wöstmann et al. 2016, 2019), we focused the spectral analysis of oscillatory power on the anticipation period in the cue–target interval. To this end, we cut out the EEG data in the time interval 0.75 to 1.5 s, which is temporally remote from cue-evoked activity and from any sound-induced activity (>1.5 s). Spectral power was obtained using FFT with multi-tapering (DPSS, discrete prolate spheroidal sequences) for frequencies 1 to 30 Hz in steps of 1 Hz with 2 Hz spectral smoothing.

Lateralization of spectral power was calculated individually for each session (1, 2), CI status (on, off), and participant for the selection of lateral targets [LI_selection_ = (Pow_select-left_ – Pow_select-right_)/(Pow_select-left_ + Pow_select-right_) and for the suppression of lateral distractors [LI_suppression_ = (Pow_suppress-left_ – Pow_suppress-right_)/(Pow_suppress-left_ + Pow_suppress-right_).

For further analyses, we followed in part a preregistered analysis plan for a previous study (https://osf.io/bv7zs;  Wöstmann et al. 2019). We averaged each lateralization index (LI_selection,_ LI_suppression_) across frequencies in the alpha band (7 to 13 Hz), separately for two sets of 12 left and 12 right hemispheric occipito-parietal electrodes (TP9/10, TP7/8, CP5/6, CP3/4, CP1/2, P7/8, P5/6, P3/4, P1/2, PO7/8, PO3/4, and O1/2).

### Statistical analyses

To estimate the reliability of audiological tests (FMST, SSQ, AMRD) from results in sessions 1 vs. 2, we used Spearman correlation coefficients. Nonparametric permutation tests were used to test for mean test-score differences in session 1 vs. 2. The reported *P*-value (denoted *p*_perm_) corresponds to the proportion of absolute values of 10 000 dependent-samples *t*-statistics computed on data with permuted condition labels exceeding the absolute empirical *t*-value for the original data.

For the EEG outcome measures in the passive listening task (GFP & ASSR), we used repeated-measures ANOVAs with the factors Session (1 vs. 2) and AM rate of interest (4 vs. 40 Hz).

For behavioral data in the spatial attention task, SRT_50_ values for individual conditions were determined and CI benefit was calculated by subtracting the SRT_50_ for CI on–CI off. CI benefits were then submitted to a repeated-measures ANOVA (including Greenhouse–Geisser correction of degrees of freedom to control for violation of sphericity) with the factors Session (1 vs. 2) and Condition (select CI-side, suppress CI-side, select non-CI-side, suppress non-CI-side), followed by post hoc nonparametric permutation tests. Only complete datasets (recordings in sessions 1 & 2) were included in this analysis. Three participants with complete datasets were excluded (ID 6, 18, 20), as they had unusually high SRTs (>+40 dB) in at least one condition, leaving *n* = 11 participants with complete datasets for the analysis of behavior in the spatial attention task.

For EEG data in the spatial attention task, we first tested for significant hemispheric modulation of LI_selection_ and LI_suppression_. To this end, we used two repeated-measures ANOVAs on average alpha (7 to 13 Hz) power across 12 left and 12 right hemispheric occipito-parietal electrodes (see above) with the factors Session (1 vs. 2), CI status (on vs. off), and Hemisphere (left vs. right). Next, we tested whether the significant hemispheric modulation of LI_selection_ would be stronger in the hemisphere on the same versus opposite side of the CI. For each participant, we calculated two alpha lateralization indices (ALIs; Wöstmann et al. 2016), one contrasting selection of ipsi- versus contralateral targets with respect to electrodes on the CI side (ALI_CI side_) and the other with respect to electrodes on the non-CI side (ALI_non-CI side_). A repeated-measures ANOVA with the factors Session (1 vs. 2), CI status (on vs. off), and Hemisphere (CI-side vs. non-CI-side) was used for statistical testing.

## Results

**Fig. 2:**
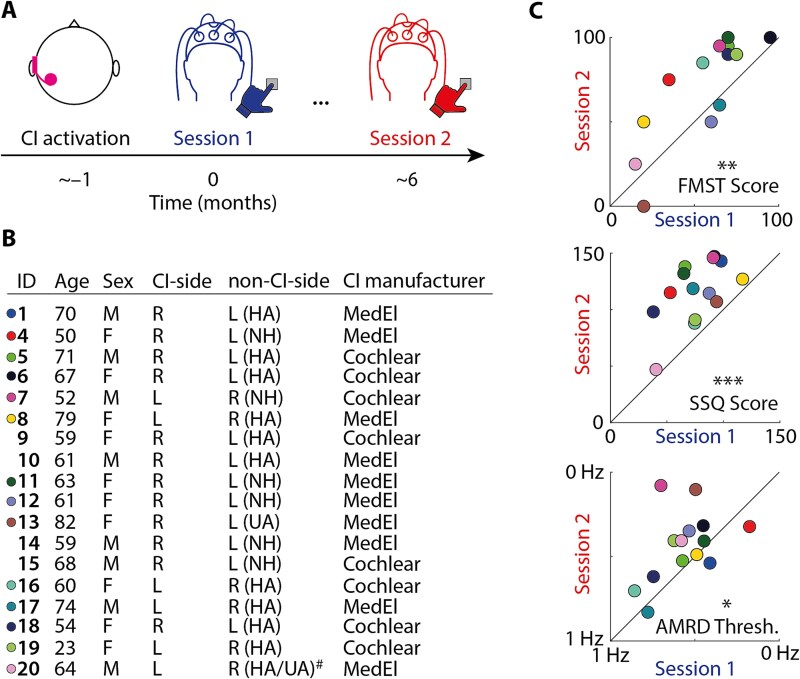
A) Schematic depiction of the general study design. Unilateral CI users completed behavioral and electrophysiological measurement sessions in the first month (session 1) and seventh month (session 2) after CI activation. (B) Participant information. Age refers to the timepoint of the first session. R, right; L, left; HA, hearing aid; NH, normal hearing (no or mild hearing loss according to WHO criteria), UA, unaided. ^#^Participant had significant deterioration of hearing on the non-CI side in-between sessions 1 & 2 and could not be adequately fitted with a hearing aid (HA in session 1; UA in Session 2). Colored circles indicate individual participants who completed both sessions (S1 & S2) and had no missing data (*n* = 14; the same color-coding is used in all forthcoming figures). C) 45-degree plots show percentage correct scores in the “Freiburg monosyllabic speech test” (FMST; top), overall score in the “speech, spatial and qualities questionnaire” (SSQ, summed over items from all three scales; middle) and thresholds in the “amplitude modulation rate discrimination task” (AMRD; bottom). Note that *x* and *y* axes for the AMRD results are reversed, such that data points above the diagonal indicate improved (ie lower) thresholds for session 2 vs. 1. ^*^P < 0.05, ^**^P < 0.01, ^***^P < 0.001.

Here, we employed an audiological and neuro-behavioral test battery in unilateral CI users at two time points: in the first and seventh months following CI activation (Fig. 2A). We hypothesized that the to-be-expected improvements in subjective and objective hearing abilities following CI activation would be accompanied by improved neural perceptual processing of sound. Furthermore, we expected that asymmetries in the neural implementation of auditory spatial attention would be explained by the side of the CI.

### Improvements in subjective and objective hearing

We administered three tests to assess speech comprehension (FMST) and subjective hearing abilities across different domains (SSQ), as well as auditory temporal discrimination ability (AMRD). To probe whether the tests are reliable, we calculated test–retest reliability, which was moderate to high (Spearman’s rho; FMST: *rho* = 0.819; SSQ: *rho* = 0.442; AMRD: *rho* = 0.392). Compared with session 1, nonparametric permutation tests revealed significant improvements in session 2 for all tests (Fig. 2C; FMST: *P_perm_* = 0.007, average performance increase = 15.36%; SSQ: *P_perm_* < 0.001, average improvement = 39.11 scores; AMRD: *P_perm_* = 0.026, average threshold decrease = 0.126 Hz).

### Passive listening: differential modulation of low- vs. high-frequency phase locking

**Fig. 3:**
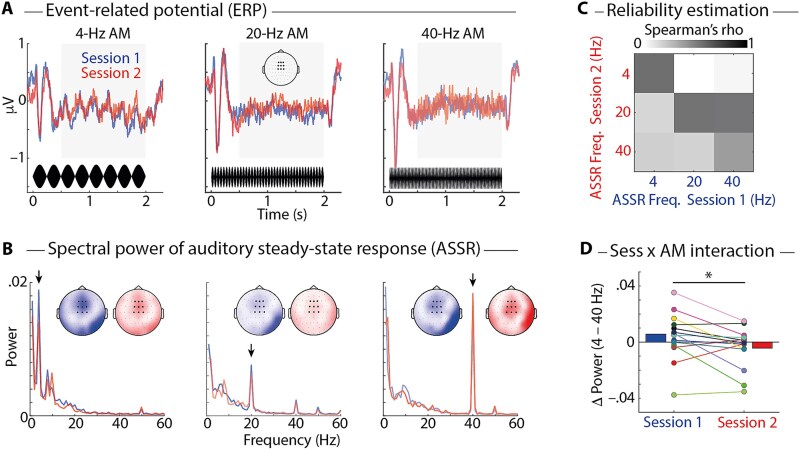
A) Event-related potential (ERP), averaged across participants in session 1 (*n* = 18, blue) and in session 2 (*n* = 14, red) and 9 fronto-central channels (highlighted in topographic map). Amplitude-modulated (AM) sound stimuli are shown in black at the bottom. B) Lines show average spectral power, calculated on the ERP in the time interval 0.5 to 2 s (gray boxes in A), averaged across 9 fronto-central channels. Arrows highlight corresponding AM rates at 4, 20, and 40 Hz. Topographic maps show spectral power (color bar limits: 0 to 0.04) at corresponding AM rates for sessions 1 (blue) and 2 (red). Single-subject ERPs and spectral power data are shown in Figs S1 and S2. C) Correlation matrix shows Spearman’s rho for ASSR peak frequencies correlated between sessions 1 & 2 (*n* = 14). D) Visualization of significant session (1 vs. 2) × AM rate (4 vs. 40 Hz) interaction. Bars show average spectral power; colored lines and dots show individual participants. ^*^P < 0.05.

CI users listened passively to AM 1000 Hz tones (Fig. 3A). Most importantly, we used AM rates of 4 vs. 40 Hz, which are thought to reveal the neural tracking of slow modulations as they occur in spoken language vs. temporal fidelity in tracking fast modulations, respectively. Additionally, we included a 20 Hz AM modulation condition.

The passive listening task served two purposes. First, an independent component analysis (ICA) could be used to identify EEG components related to the CI artifact, to auditory activity, and to other brain activity. A selection of these components could then be used for subsequent analyses. Second, we rejected all but the auditory components from these data and calculated the ASSR in the EEG to probe CI users’ auditory perceptual processing.

The ERP (Fig. 3A) to AM sounds showed obligatory onset response components (P1, N1, P2), followed by a steady-state response—the ASSR—corresponding to the AM rate. The onset response appeared to increase for higher AM rates. For statistical analysis, we quantified the overall onset response by GFP (ie the standard deviation across electrodes) in the time interval 0 to 230 ms following sound onset. There was a significant main effect of AM rate (4 vs. 40 Hz) on GFP (*F*_1, 13_ = 5.367; *P* = 0.037), indicating larger GFP for the 40 Hz compared with the 4 Hz condition (*P_perm_* = 0.029). There was neither a main effect of Session nor a Session × AM rate interaction (both *P* > 0.12).

Spectral power of the ASSR was calculated in the time interval 0.5 to 2 s following sound onset. Prominent spectral peaks corresponding to AM rates of sounds were present in these spectra (Fig. 3B). Spectral power of ASSR peak frequencies was moderately correlated across the two sessions and generally higher for corresponding frequencies (Fig. 3C), which demonstrates reliability of the ASSR measure. Critically, for the two AM rates of interest (4 & 40 Hz), there was a significant Session × AM rate interaction (Fig. 3D; *F*_1, 13_ = 6.066; *P* = 0.029). Post hoc tests revealed that for individual frequencies, there were no significant effects of Session (4 Hz: *P_perm_* = 0.127; 40 Hz: *P_perm_* = 0.459). The significant interaction was nevertheless robust and persisted when the spectral analysis was extended to incorporate the entire stimulus duration (0 to 2 s; *F*_1, 13_ = 5.380; *P* = 0.037), as well as when regressing out differences in threshold estimates (which were used to individualize stimulus presentation levels) on the CI side (*P* = 0.0345) and non-CI side (*P* = 0.0286).

Finally, we asked whether the observed AM rate (4 vs. 40 Hz) × Session (1 vs. 2) interaction was driven by a change in band-specific EEG amplitude or phase consistency, since both reflect in the spectral power of the ASSR. However, when analyzing intertrial phase coherence (which is, in theory, independent of spectral power) this effect was likely absent (*F*_1, 13_ = 0.608; *P* = 0.450; Bayes factor, *BF*_10_ = 0.398), suggesting that the AM rate effect is not solely driven by modulation of phase coherence.

### Active listening: hearing with a single-side CI benefits spatial listening

**Fig. 4:**
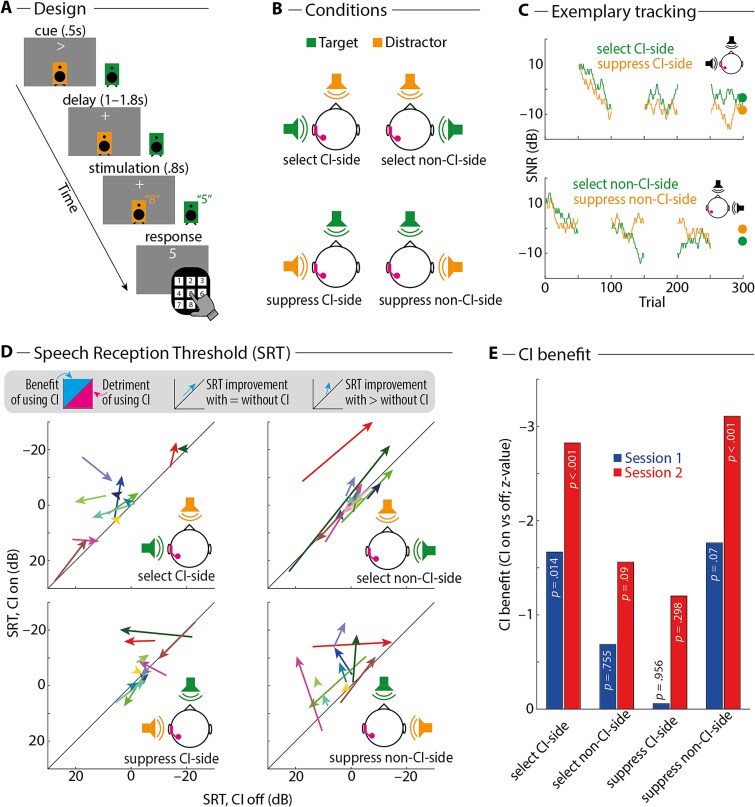
A) Design of spatial listening task. On each trial, one of two loudspeakers was the target (green; indicated by a visual cue) and the other was the distractor (orange). The task was to attend to and report the spoken number presented over the target loudspeaker. B) Experimental conditions for an exemplary participant with the CI on the left side. C) The SNR was continuously titrated throughout the task to achieve 50% task accuracy in each condition. Graphs show the adaptively tracked SNR over time for one exemplary participant (ID 19, session 1). Note that the speaker setup (front & left versus front & right) alternated in a block-wise fashion, meaning that only two of the four conditions were presented (and thus titrated) in a respective block. Large dots at the end of the tracking procedure indicate the speech reception threshold (SRT_50_), which was calculated as the average SNR across all trials of the respective condition, except for trials in the first block. D) Arrows show individual participants’ SRT for the CI switched off (*x* axis) versus on (*y* axis), pointing from the respective SRT in session 1 to session 2 (*n* = 11). More negative (ie better) SRTs are plotted upward (*y* axis) and to the right (*x* axis). Data points above the diagonal indicate better SRT when the CI was switched on versus off (ie CI benefit). E) Summary and statistical analysis of the data shown in D). Bars show *z*-values for the statistical contrast CI on versus off for individual task conditions and sessions. Negative z-values (corresponding to more negative—ie better—SRT when the CI was switched on) are plotted upward. *P*-values in bars correspond to results of nonparametric permutation tests of CI benefits against zero.

To investigate the benefits of listening with a unilateral CI in different spatial arrangements of competing target versus distractor speech, participants performed a spatial listening task with the CI turned on versus off (Fig. 4A and B). Task performance was continuously titrated using an adaptive staircase procedure, such that the SNR was adjusted for each task condition separately to arrive at 50% task accuracy (Fig. 4C). In each run of the task, participants performed six blocks, wherein three blocks presented one sound source on the left and three on the right (while the other sound source was always presented in the front). The speech reception threshold (SRT_50_) for a specific condition corresponded to the average SNR across all trials of the respective condition, except for trials in the first block. Participants performed two runs of this task, first with the CI switched on and thereafter with the CI switched off.

Overall, SRTs were lower (ie better) with the CI switched on versus off (Fig. 4D and E), which speaks to a CI benefit. Statistical analysis revealed a significant main effect of Condition on the CI benefit (*F*_1.63, 16.28_ = 4.415; *P* = 0.036), but the main effect of Session (*F*_1, 10_ = 3.722; *P* = 0.083) and the interaction of the two were not statistically significant (*F*_2, 19.97_ = 0.543; *P* = 0.589). Post hoc nonparametric permutation tests showed that a significant benefit of the CI was only observed in conditions where the implanted ear was primarily exposed to the target sound (select CI side: session 1, *P_perm_* = 0.014; session 2, *P_perm_* < 0.001; suppress non-CI side: session 1, *P_perm_* = 0.070; session 2, *P_perm_* < 0.001) but not in conditions where the implanted ear was primarily exposed to the distractor sound (select non-CI side: session 1, *P_perm_* = 0.755; session 2, *P_perm_* = 0.09; suppress CI side: session 1, *P_perm_* = 0.956; session 2, *P_perm_* = 0.298).

### Side of cochlear implant affects attentional modulation of alpha power

**Fig. 5:**
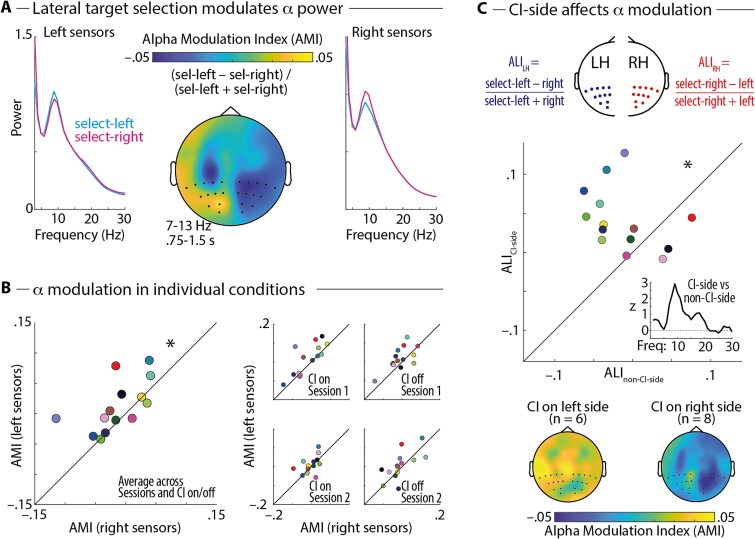
A) Spectra show oscillatory power for conditions with the attended loudspeaker on the left or right side in the time interval in between spatial cue presentation and stimulus onset, averaged across sessions (*n* = 18 in session 1, *n* = 14 in session 2) and CI status (on, off). The topographic map shows the attentional modulation index (AMI) in the alpha frequency band (7 to 13 Hz), calculated as (Power_select-left_ – Power_select-right_)/(Power_select-left_ + Power_select-right_). B) 45-degree plots show AMI averaged across posterior right- (*x* axis) versus left-hemispheric (*y* axis) sensors for participants who participated in both sessions (*n* = 14 complete datasets). Note that datapoints above the diagonal indicate the expected pattern of higher AMI values at sensors on the left versus right hemisphere. ^*^Significant main effect of hemisphere (left vs. right) on AMI in repeated-measures ANOVA (*P* < 0.05). C) Top: Illustration of the approach to calculate alpha lateralization indices (ALI) for the left (LH) and right (RH) hemispheres separately. Middle: 45-degree plot shows the ALI on the non-CI side (*x* axis) versus the CI side (*y* axis). ^*^Significant main effect of hemisphere (CI side vs. non-CI side) on ALI in repeated-measures ANOVA (*P* < 0.05). Inset shows *z*-values for the contrast of lateralization indices on the CI versus non-CI side for individual frequencies (1 to 30 Hz). Bottom: Topographic maps show AMI separately for participants with the CI on the left versus the right side.

To test CI users’ neural signatures of auditory spatial attention to competing speech stimuli, we analyzed the spatial attention-driven hemispheric lateralization of alpha oscillations (7 to 13 Hz) in the EEG. In agreement with a previous investigation with a similar paradigm in normal-hearing listeners (Wöstmann et al. 2019), we found relatively higher posterior left-hemispheric alpha power following a leftward versus rightward spatial cue, and vice versa for right-hemispheric alpha power (Fig. 5A and B). Consistent with this, a repeated-measures ANOVA revealed a main effect of Hemisphere (left vs. right) on the alpha modulation index (AMI; *F*_1, 13_ = 6.451; *P* = 0.025) but no significant main effects of Session (1 vs. 2), CI status (on vs. off), or any interaction (all *P* > 0.2).

Although we found before in a larger sample of normal hearing listeners (Wöstmann et al. 2019) a small but significant reversal of the alpha lateralization when contrasting trials with the spatial cue to the front and the distractor on the left versus right side (ie suppress-left vs. suppress-right conditions), this effect was not significant in the present dataset (*F*_1, 13_ = 0.894; *P* = 0.362).

In normal-hearing listeners, attentional modulation of lateralized alpha oscillations is typically assumed to be symmetrical between the two hemispheres. However, since spatial attention allocation in unilateral CI users is presumably affected by strong differences in hearing abilities on the left versus right side, we tested for asymmetries in hemispheric alpha lateralization. To this end, we calculated two ALIs, one contrasting attentional selection of targets on the ipsi- versus contralateral side for electrodes on the left hemisphere and another one for electrodes on the right hemisphere (Fig. 5C). A repeated-measures ANOVA revealed a main effect of Hemisphere (CI side vs. non-CI side), indicating that attentional alpha modulation was stronger in the hemisphere on the CI side (*F*_1, 13_ = 9.045; *P* = 0.01). Critically, this effect was specific to the alpha frequency range (Fig. 5C, inset). This rules out the possibility that differences in EEG data preprocessing associated with the interpolation of missing electrodes on the CI side might have introduced differential sensitivity for attentional modulation of oscillatory power between the hemispheres. Post hoc tests revealed that alpha lateralization was significant in the hemisphere on the CI-side (*P_perm_* < 0.001) but not in the hemisphere on the non-CI-side (*P_perm_* = 0.276). There were no significant main effects of Session (1 vs. 2), CI status (on vs. off), or any interaction effects (all *P* > 0.37).

In a control analysis, we found that the effect of CI side on alpha modulation could not be explained by differential alpha modulation in the left versus right hemisphere independent of CI side (nonsignificant main effect of left vs. right hemisphere: *F*_1, 13_ = 0.094; *P* = 0.764). No clear pattern of correlations between behavioral and EEG measures in the present study could be found (see Fig. S3).

## Discussion

Here, we performed a longitudinal study to investigate electrophysiological indices of auditory temporal coding and spatial attention allocation in listeners who adapt to listening with a unilateral CI. The main findings are as follows. First, 6 months post-CI activation, the ASSR relatively decreased for 4 Hz AM sounds but increased for 40 Hz sounds. Second, spatial attention induced a robust lateralization of neural alpha oscillations in unilateral CI users, which was stable over time. Third, attentional alpha modulation was stronger in the hemisphere contralateral to the nonimplanted ear, irrespective of time or of performing the task with the CI on versus off.

### Auditory perceptual processing following CI activation

Hearing with a CI has a profound impact on the sensory perception of acoustic signals. For instance, cochlear implantation is followed by increased metabolic activity in the auditory cortex (especially contralateral; Lazeyras et al. 2002). Most CI users require several months to reach maximal perceptual performance (eg Hamzavi et al. 2003; Glennon et al. 2020). Restored hearing through CIs is associated with a broad range of plastic changes in the human brain (Giraud et al. 2002). In experienced adult CI users, auditory-induced cortical activity correlates with speech perception (Green et al. 2005). Here, we tested auditory phase-locking to amplitude-modulated sounds to help unveil the underlying neural adaptation to listening with a CI.

Vocoding has been used to simulate some aspects of hearing with a CI in normal-hearing listeners (eg Green et al. 2002). Using this method, we found previously that unilateral noise-vocoding delayed the attentional modulation of neural phase–locked responses to the envelope of speech stimuli (Kraus et al. 2021), which has a modulation spectrum peaking at ~4 Hz (Ding et al. 2017). In the present study, however, we employed a passive listening task to probe the purely perceptual aspects of processing sound irrespective of attention focus.

Preferential tracking of acoustic stimuli in the theta (~4 to 7 Hz) and gamma (~30 to 50 Hz) ranges has been found before (Lakatos et al. 2005; Teng et al. 2017) and matches well with our observation of stronger phase-locking at 4 and 40 Hz compared with 20 Hz.

Differential modulation of 4 vs. 40 Hz ASSR during adaptation to a CI is presumably based on two underlying mechanisms. First, a relatively increased 40 Hz ASSR in the seventh vs. first month might suggest improved auditory temporal integration (Picton 2013). Auditory temporal processing is important for speech processing and thus for successful communication. Critically, unilateral CI users performed the passive listening task with both ears, that is, with the implanted and nonimplanted ear. It is conceivable that better integration of electric hearing (through the CI) with acoustic hearing (on the nonimplanted side) is associated with higher temporal coding acuity over time during adaptation to listening with the CI. Additionally, the increased 40 Hz ASSR might indicate increased inhibitory processing at the level of auditory cortical circuits (Toso et al. 2024).

Second, it might appear counterintuitive that the 4 Hz ASSR did not increase but decrease over time in the present study. However, larger amplitudes of cortical phase–locked responses to slow modulation frequencies have been observed before at older age (Presacco et al. 2016; Decruy et al. 2019) and in participants with hearing loss (Fuglsang et al. 2020; Orf et al. 2025). Mechanistically, a larger EEG amplitude might indicate a cortical overrepresentation, potentially compensating for weaker subcortical representations (Presacco et al. 2019). The observed decrease of the 4 Hz ASSR over time might thus indicate decreasing cortical overrepresentation and restoration of the excitation–inhibition balance. Future studies should test the hypothesis that individual differences in the balance of the 4 vs. 40 Hz ASSR are explained by the duration of deafness before CI implantation in larger samples.

### Biased auditory spatial attention in unilateral CI users

The present study probed unilateral CI users’ speech reception in different close-to-real-life arrangements of spatially competing target and distractor speech. In general agreement with previous research, we found better performance with versus without the CI (Távora-Vieira et al. 2015) and a tendency of this effect to increase over time (see also Dillon et al. 2020). Note that speech presentation levels were individually adapted to CI users’ hearing levels in both sessions (first and seventh months), which might have reduced the ability to detect longitudinal effects on behavioral performance.

Importantly, previous research in normal-hearing listeners has shown that better acoustics (ie less degradation) not only has the intended effect of improved reception of target speech. Better acoustics also favor increased interference by speech distractors (eg Ellermeier et al. 2015; Wöstmann and Obleser 2016; Wöstmann et al. 2017). Based on this, one might have expected that hearing with a unilateral CI aids target speech reception on the CI side but, at the same time, increases interference effects of speech distractors.

To the contrary, our results demonstrate a benefit of listening with a unilateral CI in all conditions, especially (and statistically significant) in those where the target speech signal is primarily presented on the CI side. Thus, a unilateral CI does not uniquely benefit processing speech on the implanted side but instead aids auditory object formation and speech perception more generally (see also Fiedler et al. 2019; Barchet et al. 2025; Chen et al. 2025).

At the neural level, in agreement with a previous study in bilateral CI users (Paul et al. 2020), we here demonstrate robust spatial attention–induced modulation of lateralized alpha oscillations in unilateral CI users. Alpha power relatively increased in the hemisphere ipsilateral to the anticipated target sound and decreased in the contralateral hemisphere. Alpha lateralization was strongest at parietal electrode sites, which is commensurate with modulation in general attention networks rather than in auditory cortex regions (Banerjee et al. 2011). The reversal of alpha power lateralization for anticipated distractors on the left versus right side, which we found before in a larger sample of normal-hearing participants (Wöstmann et al. 2019), was not observed in CI users. This, however, does not necessarily imply reduced distractor suppression in unilateral CI users, since neural signatures of distractor suppression are typically smaller in size and thus require larger samples to be detected (Wöstmann et al. 2022).

Some prior work suggests reduced attentional alpha modulation in hearing-impaired compared with normal-hearing listeners (Bonacci et al. 2019). Although the present study did not include a normal-hearing control group, the size of the observed modulation of lateralized alpha power is comparable to a previous study with normal-hearing listeners that used a similar paradigm and the same EEG recording setup (Wöstmann et al. 2019). In sum, the present results provide an important proof of principle that hemispheric lateralization of alpha oscillations serves as a robust neural signature of auditory spatial attention deployment in CI users.

Typically, the hemispheric lateralization of alpha oscillations is assumed to be largely symmetrical between the left and right hemispheres. However, interindividual variability of left- versus -right hemispheric attentional modulation of alpha power has been observed before and could partly be explained by asymmetry of subcortical structures (Mazzetti et al. 2019; Ghafari et al. 2024; Schulz and Wöstmann 2024). Here, we substantially add to this line of research and demonstrate that asymmetric hearing in unilateral CI users is associated with stronger attentional modulation of alpha oscillations in the hemisphere contralateral to the nonimplanted ear.

In theory, more pronounced spatial attention–driven alpha modulation in one hemisphere implies that the sensory input on the contralateral side is enhanced more when it is a target and/or suppressed more when it is a distractor (Strauß et al. 2014; Schneider et al. 2021). Our findings suggest that unilateral CI users apply auditory attentional processing predominantly in anticipation of auditory targets to the nonimplanted ear (note that distractors were always presented in the front in target-lateral conditions). In unilateral CI users, task-specific effects of better-ear listening have been found before (eg Williges et al. 2019), and it is likely that the nonimplanted ear is the better ear for many participants in the present study. In general, our results highlight the importance of considering bilateral auditory input in CI rehabilitation (see also Chen et al. 2025).

Critically, the observed asymmetry of alpha lateralization was stable across time (seventh vs. first month post-CI activation) and remained unmodulated by performing the task with versus without the CI. Absence of these effects could have been caused by our careful matching of task difficulty across conditions. That is, CI users’ performance was titrated to 50% correct in all conditions, meaning that task difficulty was, in theory, equal in the first vs. seventh month and with the CI on vs. off. Nevertheless, we tentatively conclude that the observed alpha asymmetry was driven by the history and presence of profound asymmetric hearing loss, rather than unilateral CI use. Future studies should test whether the asymmetric neural implementation of auditory spatial attention is present already before CI implantation and whether it declines after maximal performance in hearing with the unilateral CI is reached.

### Limitations

Of note, there are some limitations of the present study that should be considered. First, the sample size was small but the longitudinal nature of the study in part compensates for this lack in quantity by providing rich (ie “deep”) data from audiological tests and behavioral listening tasks, as well as EEG recordings in a passive listening test and in an active spatial attention task, in the first and seventh months following CI activation.

Second, the present study did not include a control group of listeners who did not receive a CI. Thus, it might be that some of the presumed CI-induced effects are partly driven by CI-independent learning mechanisms. While improving scores on audiological tests (FMST, SSQ, AMRD) might indeed be affected by learning, CI benefits in the spatial task were calculated by contrasting the CI-on with the CI-off condition in the seventh versus first month following CI activation. Thus, potential learning effects over time are being controlled for.

Third, CI conditions were tested in a fixed order (on, off) in the spatial listening task. In theory, the CI benefit might be somewhat overestimated and in part driven by adaptation to the task. However, we consider this rather unlikely since our calculation of speech reception thresholds omitted the first block for each experimental condition and thus focused on the later period where thresholds were stable.

## Conclusion

The electrophysiological evidence in our data suggests a longitudinal change in phase-locking to slower (4 Hz) versus faster (40 Hz) amplitude modulations in the first 6 months following activation of a unilateral CI. Irrespective of listening with or without the CI, unilateral CI users exhibit stronger neural attentional modulation associated with the nonimplanted side, which emphasizes the importance of listening with both ears to unilateral CI users. These data provide first steps toward a mechanistic account of auditory perceptual and attentional adaptation to listening with a cochlear implant.

## Supplementary Material

Supplementary_Materials_BimodalBenefit_bhaf244

## Data Availability

All data are available from the corresponding author upon request. A table including data for the most important behavioral and neural outcome measures is available at https://osf.io/tkurz/.
