# Supplementary material for: Neural alpha oscillations and auditory steady-state responses during adaptation to a cochlear implant
Source: Cereb Cortex. 2025 Sep 10;35(8):bhaf244. doi: 10.1093/cercor/bhaf244 (PMC12421880; doi:10.1093/cercor/bhaf244)
Supplement: Supplementary_Materials_BimodalBenefit_bhaf244 [file supplementary_materials_bimodalbenefit_bhaf244.docx]

**Supplementary Materials**

for the article

**Neural Alpha Oscillations and Auditory Steady State Responses During Adaptation to a Cochlear Implant**

Malte Wöstmann^1,2,*^, Hannah Marie Meineke^1,2,3^, Rainer Schönweiler^4^, Daniela Hollfelder^5^, Karl-Ludwig Bruchhage^5^, Anke Leichtle^5^, Jonas Obleser^1,2^

^1^ Department of Psychology, University of Lübeck, Lübeck, Germany;

^2^ Center of Brain, Behaviour, and Metabolism, University of Lübeck, Lübeck, Germany;

^3^ Department of Clinical Research, University of Southern Denmark, Odense, Denmark;

^4^ Department of Otorhinolaryngology, Phoniatrics and Paediatric Audiology, University Hospital of Schleswig-Holstein, Campus Lübeck, Lübeck, Germany;

^5^ Department of Otorhinolaryngology, Head and Neck Surgery, University Hospital of Schleswig-Holstein, Campus Lübeck, Lübeck, Germany

* Corresponding author: Malte Wöstmann, Department of Psychology, University of Lübeck, Ratzeburger Allee 160, Lübeck 23562, Germany. Email: [malte.woestmann@uni-luebeck.de](mailto:malte.woestmann@uni-luebeck.de)


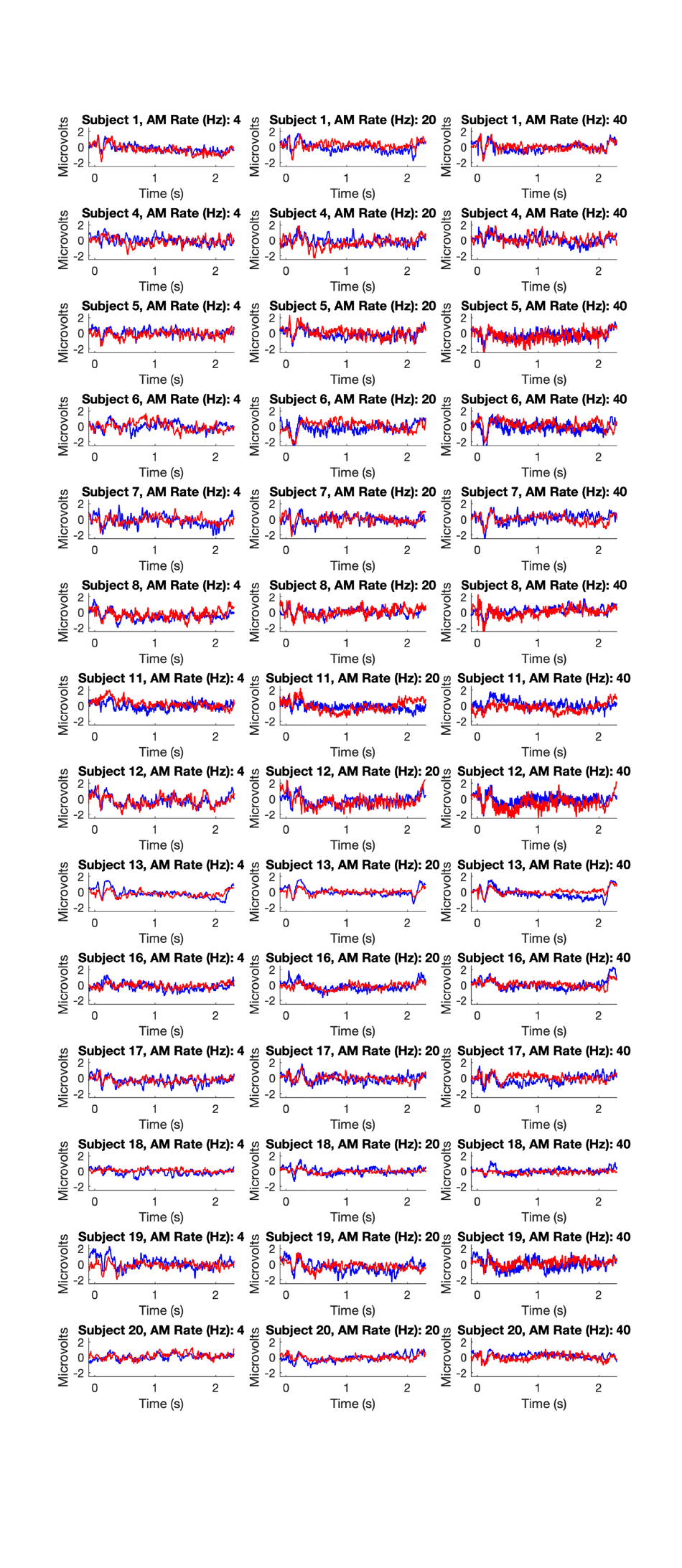


**Figure S1**. Single-subject event-related potential (ERP) to amplitude-modulated (AM) sounds (blue: session 1, red: session 2). The ERP has been averaged across 9 fronto-central channels and is shown for participants who completed both experimental sessions.


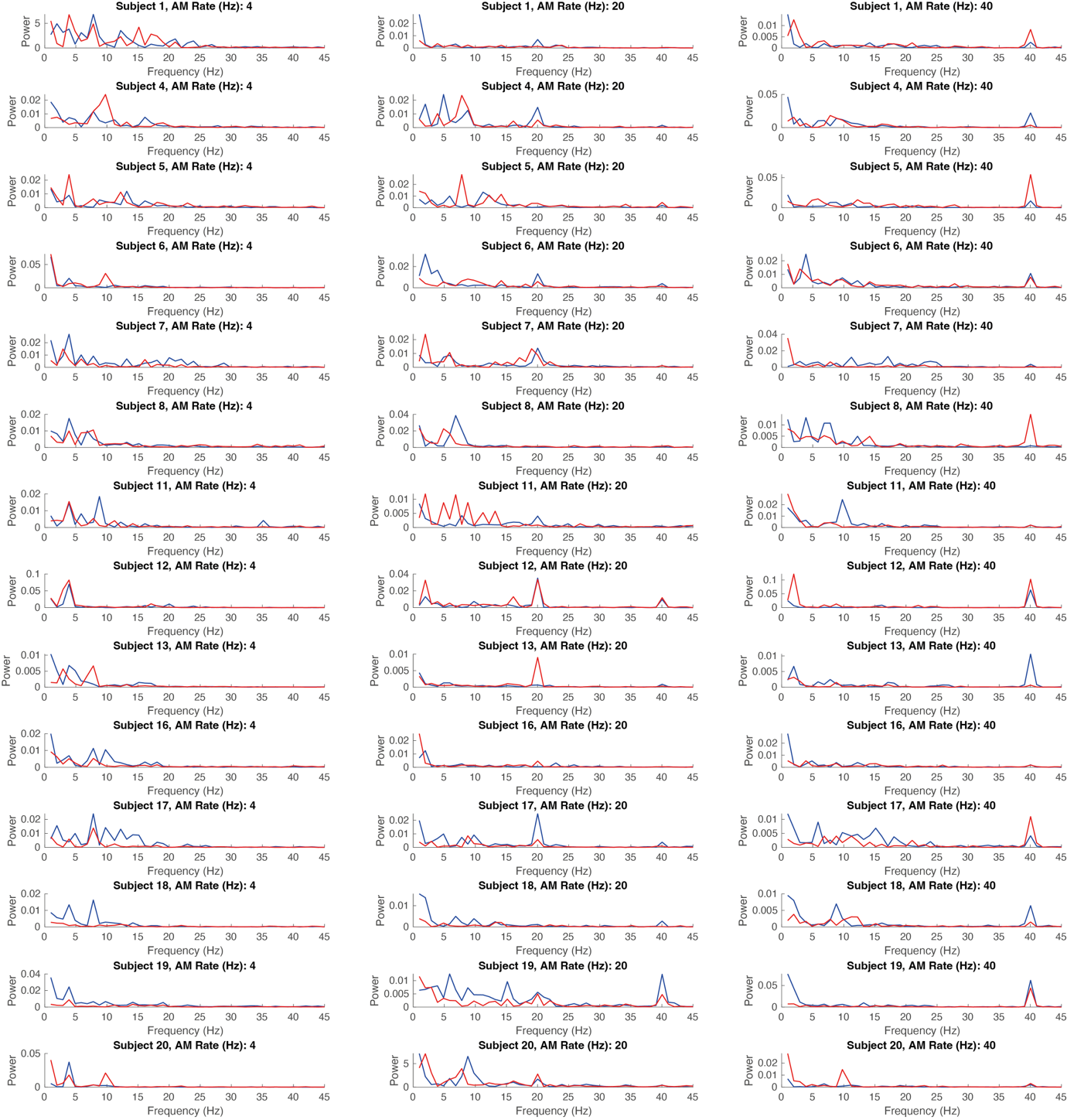


**Figure S2**. Single-subject spectral power, calculated on the ERP to amplitude-modulated (AM) sounds in the time-interval 0.5–2s, averaged across 9 fronto-central channels (blue: session 1, red: session 2). Spectra are shown for participants who completed both experimental sessions.


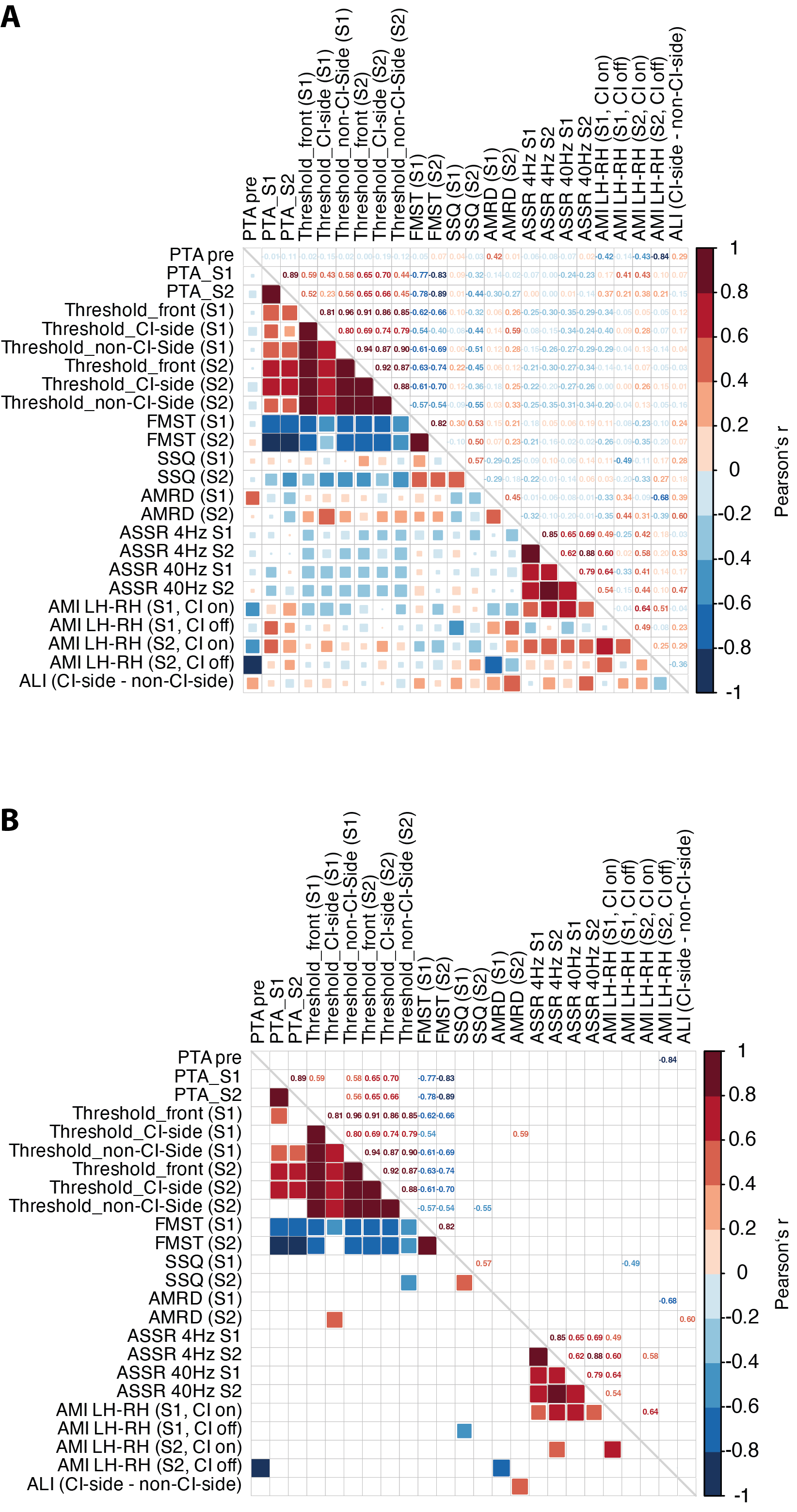


**Figure S3**. (**A**) Correlation heatmap for all variables of interest. (**B**) Same as A with all non-significant correlations (p > .05) removed. S1 = Session 1; S2 = Session 2. Note that due to the limited sample size (S1: N = 18; S2: N = 14), all correlations should be interpreted with caution.
